# Supplementary material for: Aspartate aminotransferase is required for Salmonella expansion in the inflamed gut via TCA anaplerosis
Source: bioRxiv. 2026 Feb 26:2026.02.24.707732. Preprint. [Version 2] doi: 10.64898/2026.02.24.707732 (PMC13160064; doi:10.64898/2026.02.24.707732)
Supplement: Supplement 1 [file media-1.pdf]

SUPPLEMENTARY MATERIALS

Supplementary Table 1: Bacterial strains and plasmids used in this study.

**Bacterial Strain**	**Source**	**Identification**
S. Typhimurium, IR715 ATCC 14028S Nal^R	Stojiljkovic et al.[47]	IR715
S. Typhimurium, IR715 Δ*phoN*::Tn*10d*-Cm^R	Faber et al.[48]	FF176
S. Typhimurium, IR715 Δ*phoN*::Km^R	Kingsley et al.[49]	AJB715
S. Typhimurium, IR715 Δ*aspC*::Km^R	This study	NS42
S. Typhimurium, IR715 Δ*aspC*	This study	NS43
S. Typhimurium, IR715 Δ*frdABCD*	This study	WJ28
S. Typhimurium, IR715 Δ*frdABCD*Δ*phoN*::Tn*10d*-Cm^R	This study	WJ30
S. Typhimurium, IR715 Δ*invA*Δ*spiB*	Rivera-Chávez et al.[50]	SPN487
S. Typhimurium, IR715 Δ*invA*Δ*spiB*Δ*aspC*::Km^R	This study	NS301
S. Typhimurium, IR715 Δ*invA*Δ*spiB*Δ*phoN*::Tn*10d*-Cm^R	This study	WJ52
S. Typhimurium, IR715 Δ*invA*Δ*spiB*Δ*aspC*	This study	NS311
S. Typhimurium, IR715 Δ*invA*Δ*spiB*Δ*aspC*Δ*phoN::*Km^R	This study	NS313
S. Typhimurium, IR715 Δ*sdhA*::Km^R	This study	NS314
S. Typhimurium, IR715 Δ*gltA*::Km^R	This study	NS315
S. Typhimurium, IR715 Δ*sdhA*Δ*aspC*::Km^R	This study	NS316
S. Typhimurium, IR715 Δ*gltA*Δ*aspC*::Km^R	This study	NS317
S. Typhimurium, IR715 Δ*sdhA*ΔphoN::Tn10d-Cm^R	This study	NS318
S. Typhimurium, IR715 Δ*gltA*Δ*phoN*::Tn10d-Cm^R	This study	NS319
S. Typhimurium, IR715 Δ*sdhA*Δ*aspC*Δ*phoN*::Km^R	This study	NS320
S. Typhimurium, IR715 Δ*gltA*Δ*aspC*Δ*phoN*::Km^R	This study	NS321
S. Typhimurium, IR715 WT:pWSK29 (pEV)	This study	HA109
S. Typhimurium, IR715 Δ*aspC*:pWSK29 (pEV)	This study	HA113
S. Typhimurium, IR715 Δ*aspC*:pWSK29::*aspC*	This study	HA114
**Plasmid**	**Source**	**Identification**
Plasmid: pKD46, Spec^R P_{BAD}-*gam-beta-exo oriR101 repA101*^{ts}	Datsenko and Wanner[54]	pKD46
Plasmid: pKD3, Carb^R FRT Cm^R FRT PS1 PS2 *oriR6Kγ*	Datsenko and Wanne[54]	pKD3
Plasmid: pKD13, Carb^R FRT Km^R FRT PS1 PS4 *oriR6Kγ*	Datsenko and Wanner[54]	pKD13
Plasmid: pCP20, CarbR FLP *oripSC101* CmR r*epA101^{ts}* MbeC	Datsenko and Wanner[54]	pCP20
Plasmid: pWSK29, CarbR LacZα *rep101* pSC101ori	Murray GL[55]	pEV

Plasmid: pWSK29::*aspC,* CarbR LacZα *rep101* pSC101ori *aspC*	This study	pWSK29::*aspC*

Supplementary Table 2: Primers used in this study.

Primer	Sequence
IR715_gltA_delF	GCTATTGAACTGGATGTGCTAAAAGGTACACTCGGTCAATGTAGGCT GGAGCTGCTTCG
IR715_gltA_delR	GATTTTCATGCCGTCAGTGTGCATTTCATTCCAGTGTGCATTCC GGG GATCCGTCGACC
IR715_gltA_conF	GGTCACTCTTTCACCTGTTA
IR715_gltA_conR	GCCGGAAGGTGCAGATAGAG
IR715-sdhA-del-F1	CAAACCTGTGCGCTGCTCTCGAAAGTCTTCCCGACCCGTTTGTAGG CT GGAGCTGCTTCG
IR715-sdhA-del-R1	CTGATACAGGGTATGACACACCA GTTGGCATCATCACGCATTCCGGGGATCCGTCGACC
IR715-sdhA-con-F1	GCAACGCCTCCGCATTAGGA
IR715-sdhA-con-R1	CACACCTTCGCGGCAGGAAC
aspC-del-F1	ATAGCGGATTTCCCTTCTGTAACTATAATGGACCT CGTCTGTAGGCT GGAGCTGCTTCG
aspC-del-R1	ATTGCAGGCTTTTTTATCGGTCACGCCAGTCGGCAGCTTTATTCCGG G GATCCGTCGACC
aspC-con-F1	TACTTACCGTT CTGATCAGC
aspC-con-R1	CGCTTGGCGATGATGTGAC
aspC_comp_Fwd	ATCAAGCTTATCGATACCGTC
aspC_comp_Rev	ATCGAATTCCTGCAGCCC
pWSK29_Fwd	CCGGGCTGCAGGAATTCGATTGACTACCGTTTCAACCTG
pWSK29_rev	ACGGTATCGATAAGCTTGATCTTCTTGCAAGATGAGATG
thrA_qFwd	GGCGTTCTACAGCCACTATTATC
thrA_qRev	CCGTAACAGATCGGCAAACA
metL_qFwd	GGGCGTTACTGGCTGTATAA
melL_qRev	CGCTGATCGAGAGAATCGTATC
lysC_qFwd	GGTACGCTGGTGTGCAATAA
lysC_qREv	CCTCGTGAGTGCAGCATATT
pyrI_qFwd	AACCAGTATCCTCCAGCTTTG
pyrI_qRev	TTGGCCAGCACCACATAAT
pyrB_qFwd	CAGCCGCGACGATCTTAAT
pyrB_qRev	GCTGGCGATCACTTTATGTTTC
asnA_qFwd	GAGGCTATATGGGCAGGAATTA
asnA_qRev	CAGTTCCTGACTGTGAACAAAC
IR715_qPCR_aspA_F	TCGTAACGGTGGTGTCGTTA
IR715_qPCR_aspA_R	CTGTGGCGCATATGTTATGG
IR715_qPCR_aspC_F	CAGACCGCTGAATGAGAACA
IR715_qPCR_aspC_R	GCCATTCGCGCTAACTACTC
IR715_qPCR_dcuA_F	GATGACCCTGGATGACGCTA
IR715_qPCR_dcuA_R	AAACCGCTGATGAACACCAC
K1	CAGTCATAGCCGAATAGCCT

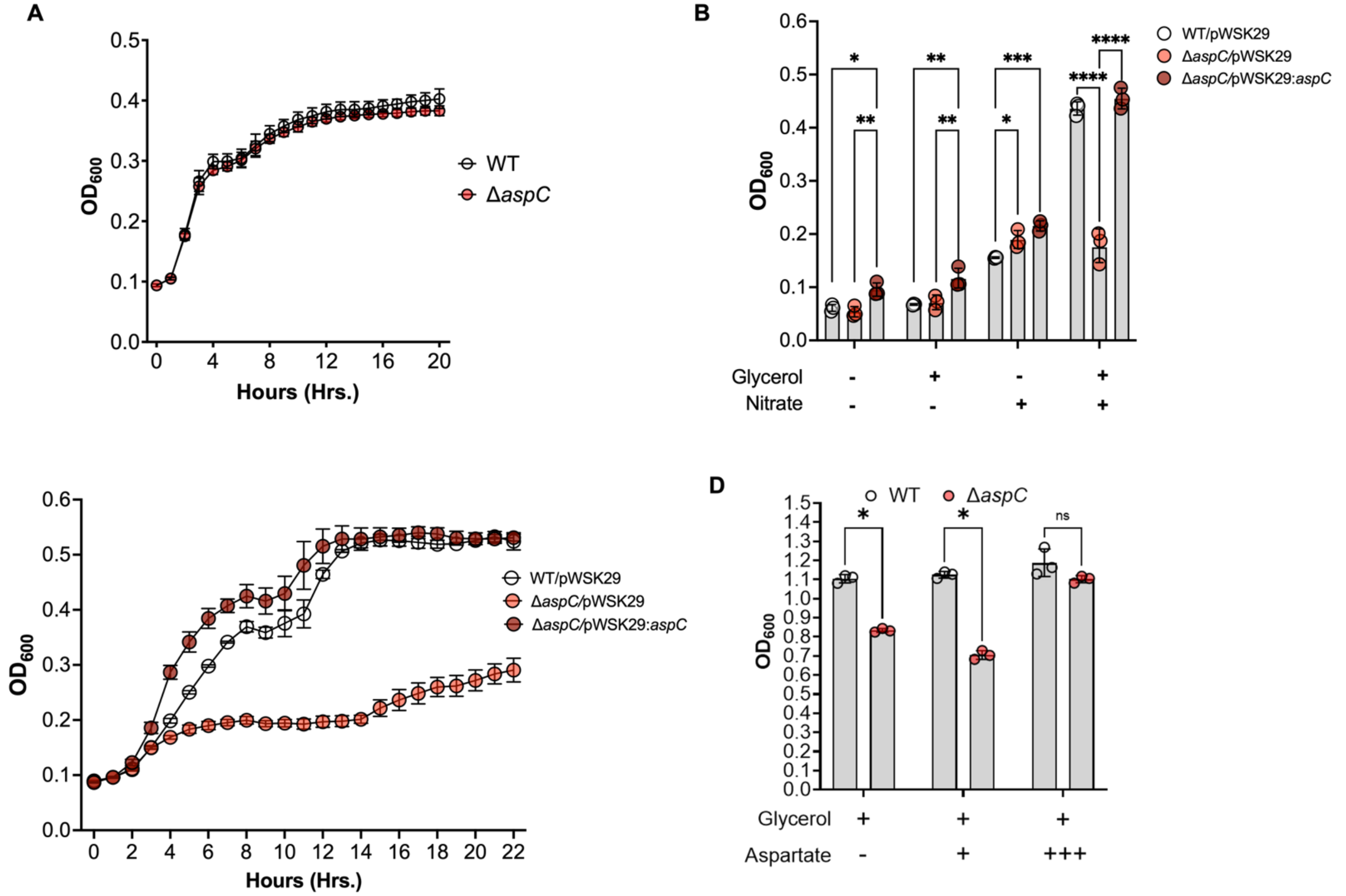

Figure S1: Deletion of ∆*aspC* does not confer a growth defect compared to WT when cultured anaerobically in LB broth but does under hypoxia in minimal media. Related to Figure 4. (A) Growth of WT and *aspC*-deficient *S*. Tm (∆*aspC*) anaerobically in LB. (B) Growth kinetics of WT (WT/pWSK29), *aspC*-deficient (∆*aspC*/PWSK29), and complemented *aspC*-deficient *S.* Tm (∆*aspC*/pWSK29*:aspC*) under anaerobic conditions in NCE media supplemented with glycerol (40 mM) and/or nitrate (40 mM) were measured at 18 hours post-inoculation. (C) Growth kinetics of WT (WT/pWSK29), *aspC*-deficient (∆*aspC*/PWSK29) and complemented *aspC*-deficient *S.* Tm (∆*aspC*/pWSK29*:aspC*) under anaerobic conditions in NCE media supplemented with glycerol (40 mM) and nitrate (40 mM). (D) Growth of WT and *aspC*-deficient *S*. Tm ((∆*aspC*) under hypoxic (0.8% O_2) NCE minimal media supplemented with Glycerol and (+) 0.1mM or (+++) 10mM Aspartate with Glycerol. N=3 per condition. Geometric mean and Geometric SD. *, p<0.05; **, p<0.01; ***, p<0.001 using 2-way ANOVA (B) and multiple unpaired t-test (D).

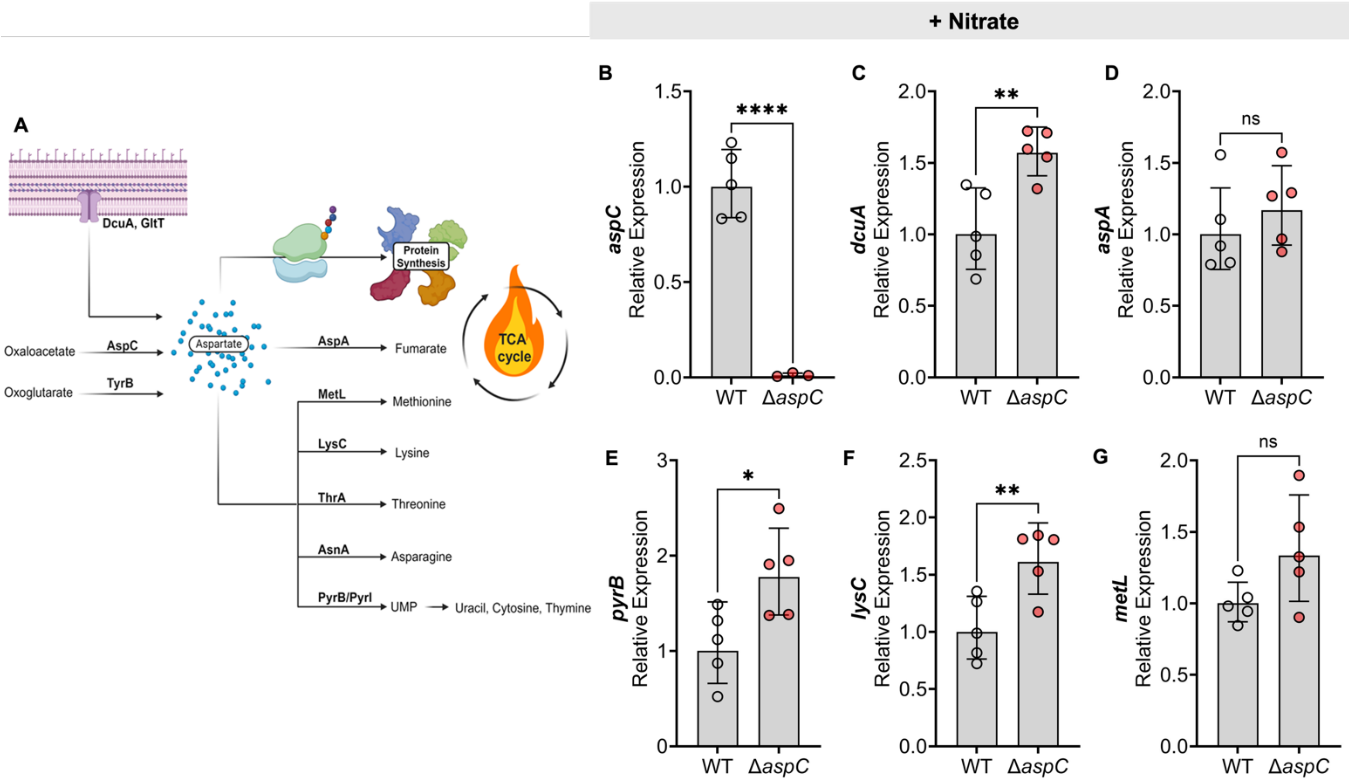

Figure S2: AspC-deficiency alter central metabolism of *S.* Tm during anaerobic nitrate respiration. Related to Figure 5. As aspartate can fuel several other metabolic processes, we investigated the expression of genes involved in aspartate utilization in conditions supplemented with nitrate, in which we observed a significant growth and fitness defect of the *aspC*-deficient *S.* Tm (Δ*aspC*) compared to the WT. (A) Schematic for the central role of aspartate in *S.* Tm metabolism. (B-G) Gene expression of WT and Δ*aspC* grown anaerobically under nitrate respiration (40 mM nitrate) with glycerol (40 mM) as the sole-carbon source. (B) *aspC,* (C) *dcuA,* (D) *aspA,* (E) *pyrB***,** (F) *lysC***,** (G) *metL***.** N=5 per condition, Geometric mean and Geometric SD. *, p<0.05; **, p<0.01; ***, p<0.001; ****, p<0.0001 using unpaired t-test.

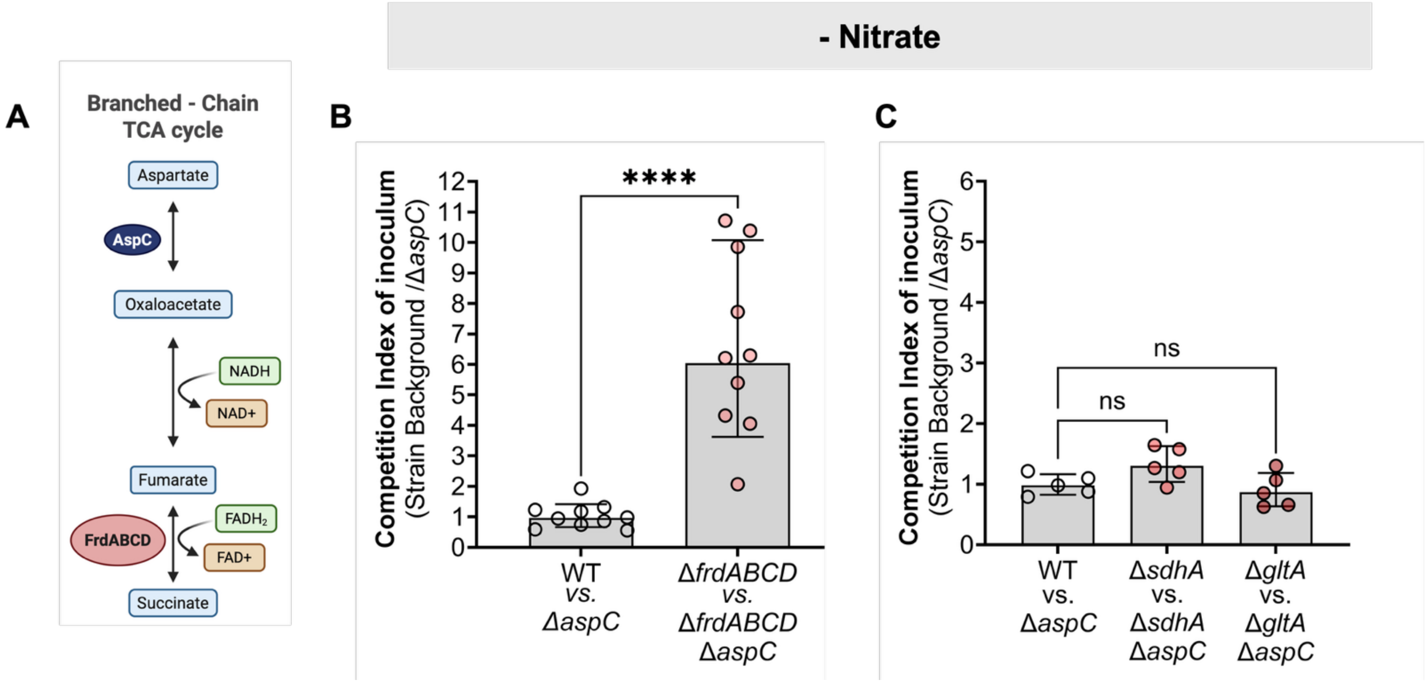

Figure S3: Deletion of *frdABCD* but not *sdhA* or *gltA* significantly increases the fitness defect of *aspC*-deficient *S.* Tm during *in vitro* growth in mucin media alone. Related to Figure 5. (A) Competitive index of inoculum for loss of *aspC (ΔaspC)* in WT and *frdABCD*–deficient *(ΔfrdABCD) S*. Tm backgrounds anaerobically grown in mucin media for 18 hours in the absence of nitrate**.** (B) Competitive index of inoculum for loss of *aspC (ΔaspC)* in WT and *gltA (ΔgltA) or sdhA (ΔsdhA)*–deficient backgrounds anaerobically grown in mucin media for 18 hours in the absence of nitrate**.** N=5-10 per condition. Geometric mean and Geometric SD. ****, p<0.0001 using unpaired t-test.
